# Non-Leukemic Granulocytic Sarcoma Presenting with Multiple Skin Nodules and a Retroperitoneal Mass

**DOI:** 10.4274/tjh.2011.0035

**Published:** 2013-03-05

**Authors:** Merve Pamukcuoğlu, Kadir Acar, Nalan Akyürek, Gülsan Türköz Sucak

**Affiliations:** 1 Gazi University University, School of Medicine, Department of Hematology, Ankara, Turkey; 2 Gazi University University, School of Medicine, Department of Pathology, Ankara, Turkey

**Keywords:** Granulocytic sarcoma, Skin, Monocytic differentiation

**To the Editor**

Here we present a case of multiple granulocytic sarcomas with monocytic differentiation involving the skin and retroperitoneal area. 

A 19-year-old man presented with the complaints of generalized arthralgia and fixed non-tender nodular lesions (Figure 1). Physical examination revealed generalized immobile, painless violaceous erythematous nodules on his back, bilateral arms and legs, and joints. The largest nodule was 3x2 cm, located on his right posterolateral upper leg. Abnormal laboratory values included hemoglobin of 84 g/L, hematocrit of 27.2%, erythroid sedimentation rate of 58 mm/h, and lactate dehydrogenase of 613 IU/L. Blood smear revealed hypochromia and microcytosis, and bone marrow was normocellular without blasts. However, monocyte and macrophage marker CD163 was positive in 10%-15% of the bone marrow cells. The pathological examination of the nodular lesions revealed medium-sized, irregular-shaped blastic cells with frequent mitotic activity, which infiltrated the dermis. The tumor tissue contained tingible body macrophages with nuclear debris. In immunohistochemical study, these cells were positive for CD163, lysozyme, CD4, and CD43, while CD117, MPO, CD68, langerin, CD1a, S–100, CD35, CD21, clusterin, fascin, factor 13a, CD123, TCL–1, TdT, CD3, CD20, and CD30 were all negative. The Ki–67 proliferation index of the neoplastic cells was 80%. In situ hybridization study with the EBER probe for Epstein-Barr virus was negative. 

Abdominal and thorax computerized tomography (CT) was performed, which revealed a right retroperitoneal mass with blurred margins. A CT-guided biopsy was performed. The biopsy showed pleomorphic cell infiltration with large mononuclear and binuclear cells with clear nucleoli, which were CD163-positive, CD117-negative, and focal MPO-positive. These atypical pleomorphic, monocytic-histiocytic cells did not have blastic morphology. A high dose of cytosine-arabinoside and idarubicin was started. The nodular lesions disappeared 20 days after starting chemotherapy. Arthralgia, dyspnea, and respiratory distress developed consequently. Right pleural effusion was demonstrated in the chest X-ray. The cytological examination of the pleural fluid revealed blastic infiltration. Since the retroperitoneal mass persisted in follow-up abdominal CT, a new chemotherapy regimen containing fludarabine, cytosine arabinoside, and idarubicin was started. However, pleural effusion persisted and required Pleurocan evacuation. Methotrexate (Mtx) was given intrapleurally accompanied with systemic high-dose Mtx. Tumor lysis syndrome developed 28 days after this regimen and required intubation; the patient died due to cardiac arrest 3 days later. 

Granulocytic sarcoma (GS) is the tumoral infiltration of extramedullary sites with immature myeloid precursor cells; it is associated with 3%-8% of acute myeloid leukemia (AML) cases and rarely with chronic myeloproliferative disorders [1]. Monocytic differentiation of GS is seen mostly in AML M4 and M5 subtypes according to the French-American-British (FAB) classification [2]. Any extramedullary anatomic site may be involved with GS and it might rarely precede but usually occurs simultaneously with acute leukemia. CD163 positivity is controversial in GS. While Lau et al. claimed that CD163 is a highly specific marker for cells of monocyte/macrophage lineage, they did not demonstrate CD163 positivity in GS in their series [2]. Backe et al., however, demonstrated CD163 positivity in myeloid leukemias with monocytic differentiation [3], while Benet et al. demonstrated CD163 positivity in 52% of their cases of leukemia cutis [4].

The prevalence of skin involvement in myeloid malignancies is approximately 3%, and it is usually associated with cases with monocytic differentiation. It is difficult to identify the origin of the tumor when it is not associated with bone marrow infiltration, namely “aleukemic leukemia cutis” [4]. In a series of 173 patients with leukemia cutis, 7.5% of the patients with leukemic skin infiltration had no underlying myeloid neoplasm [4). Skin is one of the most common sites of involvement in GS, with approximately one-quarter of patients with GS having skin involvement [4]. The presented patient had arthralgia and multiple granulocytic nodules on various parts of the body and joints without bone morrow involvement. The morphologic appearance of the skin lesions was typical for leukemia cutis in its color and nodular form, as is immunohistochemistry with monocytic differentiation. Nevertheless, most of the aleukemic GS of skin is known to be associated with infiltration with cells of monocytic differentiation [5].

In conclusion, myeloid leukemias might present with granulocytic sarcomas, particularly in skin without bone marrow involvement. Multiple skin lesions of nodular pattern with violaceous color might suggest an underlying myeloid neoplasm. The positivity of monocytic markers such as CD68 and CD163 is typical for leukemia with a predilection to the skin. Patients with aleukemic granulocytic sarcoma might have a very aggressive course, with evolution to tumor lysis syndrome.

**Conflict of Interest Statement**

The authors of this paper have no conflicts of interest, including specific financial interests, relationships, and/ or affiliations relevant to the subject matter or materials included.

## Figures and Tables

**Figure 1:**
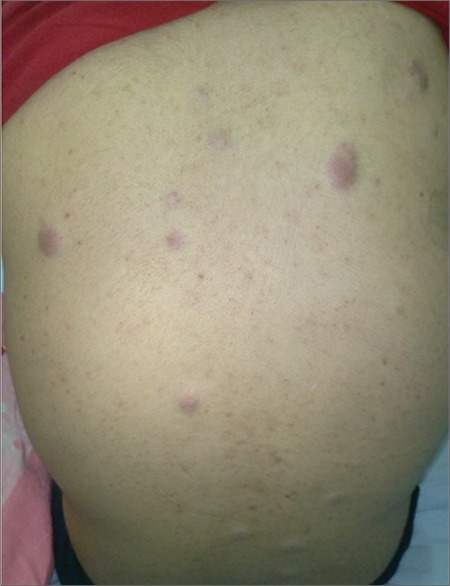
Multiple violaceous skin nodules.

## References

[1] Srinivasan B, Ethunandan M, Anand R, Hussein K, Ilankovan V (2008). Granulocytic sarcoma of the lips: report of an unusual case. Oral Surg Oral Med Oral Radiol Endod.

[2] Lau SK, Chu PG, Weiss LM (2004). CD163: A specific marker of macrophages in paraffin- embedded tissue samples. Am J Clin Pathol.

[3] Backe E, Schwarting R, Gerdes J, Ernst M, Stein H (1991). Ber-MAC3: New monoclonal antibody that defines human monocyte/macrophage differentiation antigen. J Clin Pathol.

[4] Benet C, Gomez A, Aguilar C, Delattre C, Vergier B, Beylot-Barry M, Fraitag S, Carlotti A, Dechelotte P, Hospital V, d'Incan M, Costes V, Dereure O, Ortonne N, Bagot M, Laroche L, Blom A, Dalac S, Petrella T (2011). Histologic and immunohistologic characterization of skin localization of myeloid disorders: a study of 173 cases. Am J Clin Pathol.

[5] Ohno S, Yokoo T, Ohta M, Yamamoto M, Danno K, Hamato N, Tomii K, Ohno Y, Kobashi Y (1990). Aleukemic leukemia cutis. J Am Acad Dermatol.

